# Persistent post-traumatic headache: a migrainous loop or not? The preclinical evidence

**DOI:** 10.1186/s10194-020-01135-0

**Published:** 2020-07-14

**Authors:** Silvia Benemei, Alejandro Labastida-Ramírez, Ekaterina Abramova, Nicoletta Brunelli, Edoardo Caronna, Paola Diana, Roman Gapeshin, Maxi Dana Hofacker, Ilaria Maestrini, Enrique Martínez Pías, Petr Mikulenka, Olga Tikhonova, Paolo Martelletti, Antoinette MaassenVanDenBrink, On behalf of the European Headache Federation School of Advanced Studies (EHF-SAS)

**Affiliations:** 1 Health Sciences Department, University of Florence and Headache Centre, Careggi University Hospital, Florence, Italy; 2 Division of Vascular Medicine and Pharmacology, Department of Internal Medicine, Erasmus University Medical Center, Dr Molewaterplein 50, 3015 GE Rotterdam, The Netherlands; 3 Pain Clinic Unit, Department of Anesthesiology, Pirogov City Clinical Hospital, Moscow, Russia; 4 Campus Biomedico University, Rome, Italy; 5 Neurology Department, Hospital Universitari Vall d’Hebron, Barcelona, Spain; 6 Child Neuropsychiatry Unit, Department of PROMISE, University of Palermo, Palermo, Italy; 7 Department of Neurology and Manual Medicine, Pavlov First Saint-Petersburg State Medical University, Saint-Petersburg, Russia; 8 Department of Neurology, Headache Centre, Charité Universitatsmedizin Berlin, Berlin, Germany; 9 Department of Human Neurosciences, Sapienza University of Rome, Rome, Italy; 10 Neurology Department, Hospital Clínico Universitario of Valladolid, Valladolid, Spain; 11 Department of Neurology, Third Faculty of Medicine, Charles University and University Hospital Kralovske Vinohrady, Prague, Czech Republic; 12 Department of neurology, Kazaryan Clinic of Epileptology and Neurology, Moscow, Russia; 13 Department of Clinical and Molecular Medicine, Sapienza University of Rome, Rome, Italy

**Keywords:** Animal models, Headache, Migraine, Pain, Traumatic brain injury

## Abstract

**Background:**

According to the International Classification of Headache Disorders 3, post-traumatic headache (PTH) attributed to traumatic brain injury (TBI) is a secondary headache reported to have developed within 7 days from head injury, regaining consciousness following the head injury, or discontinuation of medication(s) impairing the ability to sense or report headache following the head injury. It is one of the most common secondary headache disorders, and it is defined as persistent when it lasts more than 3 months.

**Main body:**

Currently, due to the high prevalence of this disorder, several preclinical studies have been conducted using different animal models of mild TBI to reproduce conditions that engender PTH. Despite representing a simplification of a complex disorder and displaying different limitations concerning the human condition, animal models are still a mainstay to study in vivo the mechanisms of PTH and have provided valuable insight into the pathophysiology and possible treatment strategies. Different models reproduce different types of trauma and have been ideated in order to ensure maximal proximity to the human condition and optimal experimental reproducibility.

**Conclusion:**

At present, despite its high prevalence, PTH is not entirely understood, and the differential contribution of pathophysiological mechanisms, also observed in other conditions like migraine, has to be clarified. Although facing limitations, animal models are needed to improve understanding of PTH. The knowledge of currently available models is necessary to all researchers who want to investigate PTH and contribute to unravel its mechanisms.

## Background - definition, epidemiology, why animal models are needed?

Animal models have been used for centuries in biomedical research to increase scientific knowledge and, despite increasing ethical concerns, nowadays remain one of the main tools to improve the understanding of diseases [1–3] Their use is based on the anatomical, physiological, and pathological similarities between humans and different species, especially mammals [4, 5].

Investigations in the field of pain in humans, considering both the subjective nature of the phenomenon and the numerous ethical issues, have often been limited and, consequently, the use of animal models has been larger than in other scientific areas throughout history [6]. Nowadays, pain animal models are a matter of debate, and their replacement in favor of more extensive tests in humans or the use of alternative models has been promoted [4, 7, 8] but remains a utopia [4]. The absence of biochemical or genetic biomarkers that help to predict pain occurrence and evolution, and response to treatments, favor animal models remaining a mainstay in pain science to test hypotheses and finally to improve the health of humans and animals [6]. This fact is no doubt applicable also to post-traumatic headache (PTH).

According to the International Classification of Headache Disorders 3 [ICHD-3], PTH attributed to traumatic head injury is a secondary headache that develops within 7 days after head or neck trauma (or after regaining consciousness following the trauma) [9]. Importantly, in all patients a traumatic injury to head or neck precedes the onset of PTH. However some patients may have pre-existing primary headaches; therefore, the ICHD-3 criteria require a significant worsening in close temporal relation to the trauma. PTH is acute when lasting less than 3 months, and persistent when lasting more than 3 months. PTH can be attributed to a mild, moderate or severe traumatic brain injury (TBI), whiplash, or craniotomy. However, we will focus on PTH attributed to TBI. The most common causes of mTBI leading to PTH include traffic accidents and falls; to a much lesser extent, also violence and sport injuries are reported [10]. PTH may be the only symptom following TBI, but cognitive, mood, sleep, and autonomic symptoms can also be present [11, 12]. In a large cohort study about the occurrence of post-concussion symptoms after complicated and uncomplicated mild traumatic brain injury (mTBI) it was seen that headache could be present at three and six months post-injury, as well as dizziness, noise sensitivity, fatigue/tiring more easily, feeling depressed/tearful, feeling frustrated or impatient, forgetfulness/poor memory, poor concentration, taking longer to think and restlessness [13].

Epidemiological studies of PTH have shown conflicting results, which might be due to methodological differences such as classification of PTH, the time point of evaluation, or selection criteria [14]. Nevertheless, due to the high incidence of head trauma, PTH is an important secondary headache disorder [15, 16]*.* A recent multicentric study noted that mild or worse PTH after uncomplicated mTBI (no intracranial abnormalities in CT scans) was present in 30% of patients at 3 months and in 27% at 6 months [13]. In complicated mTBI (i.e. mTBI with intracranial abnormalities in CT scans), the incidence of PTH was only slightly higher: 33% of patients at 3 months and 30% of patients at 6 months [13]. According to the Akershus Study on 30,000 persons aged 30–44 years the 1-year prevalence of chronic posttraumatic headache was 0.21% [17], another study noted that the lifetime prevalence was 4,7% in men and 2,4% in women [15].

There is currently not enough evidence to support any clear cut risk factors worth presenting as established [18]. However, some findings may be considered as initial evidence. It has been reported that younger age, female sex. and a pre-injury history of headache are significantly related to a higher risk of developing PTH [19–21]. Females were more likely to report any headaches over a 12 month-period after injury than males, and individuals with a history of headache were more likely to report headaches compared to those without [19]. Moreover, a Danish study found that women were more likely to develop chronic PTH than men, but not other post-traumatic symptoms [20]. However, it is worth noting that in some longitudinal studies, no association has been found between sex and headache after traumatic injury [10, 22]. In a study in war veterans of Iraq and Afghanistan, the prevalence of PTH was slightly higher among men than women [23].

Most studies have reported that the most frequent clinical presentation is migraine-like headache [24–26], others have reported a higher incidence of tension-type-like headache [27, 28] or more than one type of headache in a patient [10]. To a much lesser extent, other types of headaches such as cluster-like headache, cervicogenic-like headache, or unclassifiable headache are reported [10, 24, 25, 27].

Currently, animal models of mTBI and concussion are being used for studying PTH. These models, which consist of experimental penetrating or nonpenetrating head injury, are utilized for studies that, for obvious ethical reasons, cannot be performed in humans. In particular, graded cortical contusions or subcortical injuries are produced by precisely controlled brain deformations in order to study the influence of contact velocity and level of deformation on the anatomic and functional severity of TBI. According to current knowledge, the changes in the physiology of the brain, brainstem, and spinal cord following pathologic phenomena result to be a function of both contact velocity and the amount of tissue deformation [29]. Despite being a simplification of complex disorders, animal models are therefore necessary and can provide us with valuable insights into pathophysiology and possible treatment of PTH [24].

## Main text

### Animal models of PTH

**Fig. 1:**
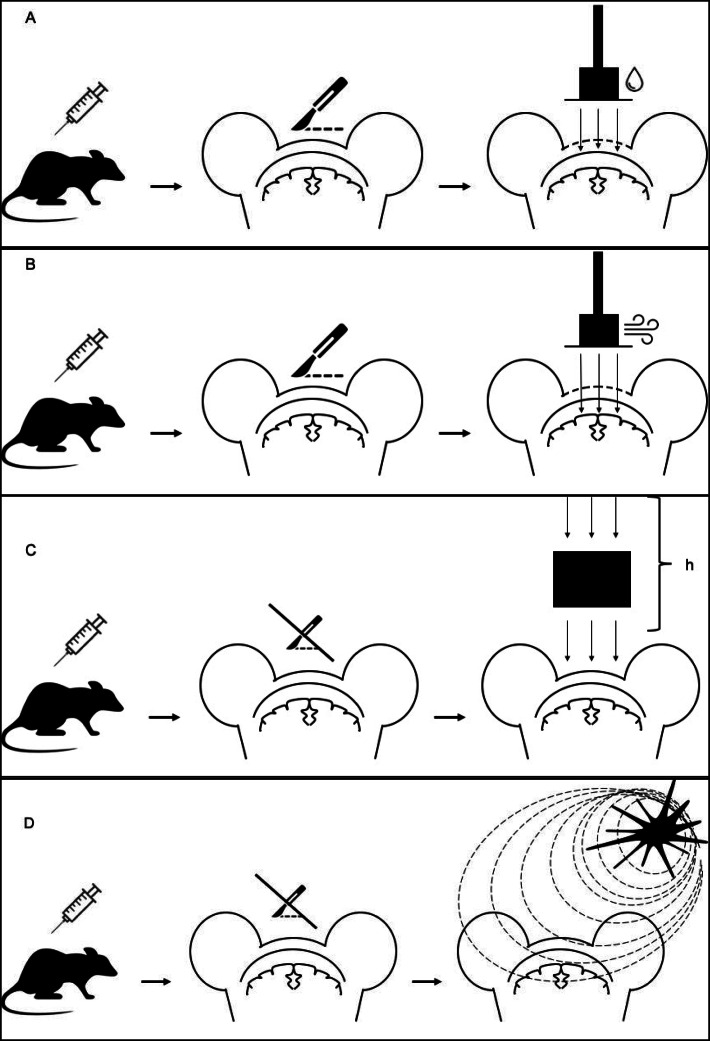
Most common animal models of traumatic brain injury (TBI). ***Penetrative injuries (a,b).*** The lateral fluid percussion, LFP injury (**a**) is generated by a pulse of pressurized fluid to the intact dura mater through a craniotomy. The controlled cortical impact, CCI injury (**b**) is done by means of a pneumatic impactor that hits the cortex through a craniotomy. In penetrative injuries the dura may be damaged, especially in controlled cortical impact models. ***Non-penetrative injuries (c,d).*** In the weight-drop injury model (**c**) a weight falls from a predetermined height (**h**), hitting the head. In the blast injury model (**d**), the animal is exposed to an explosive detonation. Non-penetrative models are the most used to study post-traumatic headache. In TBI models, animals are previously anesthetized

In the last years, different animal models of mTBI have been used to reproduce the traumatic events preceding PTH and thus allowing the study of this condition and its associated symptoms (Fig. 1). However, it is necessary to specify that, up to date, there are no well-established models of PTH, as all the models are related to TBI.

Models of mTBI can be divided into penetrative and non-penetrative injuries. Among penetrative injuries, two models have been developed: the Controlled Cortical Impact injury (CCI) and the Lateral Fluid Percussion injury (LFP). CCI is realized using a pneumatic impactor that hits the cortex through a unilateral craniotomy [29], whereas LFP induces brain injury by generating a pulse of pressurized fluid to the intact dura mater through a craniotomy [30] (Table 1). Among non-penetrative injuries, in the weight-drop injury model a projectile-shaped weight with a smooth surface falls from a predetermined height and hits the head (fixed or not) of an anesthetized animal [31], while in the blast injury model, the animal is usually exposed to an explosive detonation [32] (Table 1, Fig. 1). It is worth noting that penetrative injury models are the most used in the headache field, although the non-penetrative weight drop model is the most relevant from a translational point of view [1].

**Table 1:** Model specific pros and cons of experimental models

Model	Pros	Cons
***Penetrative injuries***		
**Controlled cortical impact (CCI) injury**	- It affords control all over biomechanical parameters. - It lacks the risk of rebound or second-hit injury, as can happen with gravity-driven devices. - The dura mater is not pierced during the procedure - Allows researchers to quantify the relationship between measurable engineered parameters (e.g., force, velocity, depth of tissue deformation) and the extent of (either functional and/or tissue) impairment. - Injury can be controlled to produce a range of injury magnitudes, allowing gradable functional impairment, tissue damage, or both. - It is the best characterized model of PTH in rodents	- The need for a craniotomy contrast with the fact that the majority of PTH cases results from non-penetrative head injuries. - Craniotomy per se can produce inflammation and sensitization of meningeal afferents, thus proper selection of controls is mandatory. - Pharmacological intervention is not applicable.
**Lateral fluid percussion (LFP) injury**	- It produces a robust and reproducible behavioral phenotype (cephalic hypersensitivity) that is suited to the study of PTH in rodents. - It exhibits predictive validity and the reliability of the technique enables the evaluation of various pharmacological and genetic manipulations before or after the induction of injury.	- It does lack translational relevance primarily due the invasive craniotomy required, and subsequent compromise of dural integrity even before the injury is applied. - Still limited application. - As only hind paw allodynia was evaluated, it is difficult to determine the relevance to PTH.
***Non-penetrative injures***		
**Weight-drop injury**	- It produces a robust behavioral phenotype, with strong translational relevance making it eminently suitable for the study of PTH. - The severity of the injury can be modified by adjusting the weight and height from which it is dropped. - Variations exist as to whether the skull or scalp are kept intact during the procedure - Increased translational value as the location and magnitude of the head traumas that lead to PTH are highly variable.	- The variability of the procedure itself; may hardly ensure that hits are identical to each other and also to avoid rebound second hits - Pharmacological intervention is not applicable.
**Blast injury**	- The experimental setup allows for the exposure of animals to a “pure” blast event without reflected shock fronts from the ground or other surfaces.	- Issues surrounding standardization and implementation are a concern and hindrance for the widespread uptake of blast injury-related models

From a clinical standpoint, following mTBI, patients may present PTH with clinical features frequently resembling migraine or tension-type headache [33]. Importantly, not all people after mTBI suffer from PTH, and the lack of disease development may also occur in animals used to model it. Considering that animals can not verbally refer pain, in vivo models have focused on pain-related behavioral phenotypes as indicative of headache. The onset of pain-related behaviors not previously present just after mTBI suggests that PTH may be studied in animal models as well. The frequent PTH phenotype similar to migraine and the existence of animal pain-related behaviors aimed to study this primary headache, probably explain why certain behavioral tests have been exploited in the study of PTH [34]. It could be argued that observed features are just modeling TBI without any PTH, and this can not be excluded at all. However, some initial evidence just points to the other direction. For instance, cranial hypersensitivity has been assessed as a marker of cephalic cutaneous allodynia, a common migraine feature [35] that is reported in PTH patients as well [36], reflecting sensitization of the trigeminal system [37]. The behavioral test used to evaluate the mechanical pain hypersensitivity in rodents consists in the application of calibrated or electronic von Frey monofilaments to their head, usually in the periorbital region, assessing head retraction as a response [38]. This test is widely used and has been applied to different animal models, such as CCI [39] and mild-closed head injury (mCHI ) [40], a type of weight-drop injury.

Von Frey filaments can be used to assess also hind paw hypersensitivity, which represents a marker of extra-cephalic allodynia, reflecting central sensitization at a higher level than the trigeminal nucleus caudalis. Nevertheless, this symptom is less commonly described in headache patients, including migraine [37], and in PTH, it has been reported immediately following mTBI in nonfixed head weight-drop injury [41] but not in fixed head weight-drop models [34], making its interpretation difficult in translational terms. If on one side, hind paw hypersensitivity cannot be considered specific for PTH, on the other, it can still be an additional tool to evaluate if PTH involves central sensitization. In this context, provocation studies assessing pericranial and hind paw hypersensitivity after administration of glyceryl trinitrate (GTN) or bright light stress (BLS) have been used to study susceptibility to headache triggers and therefore to investigate the presence of persistent central sensitization, a hallmark of headache chronification, in both migraine [42] and PTH models [34, 41].

However, not all PTH patients present allodynia, and other features have been investigated, using other evoked or spontaneous behavioral models. The multidimensional nature of PTH pain can be studied observing the spontaneous locomotor and exploratory activities in an open field environment, associating headache-behaviors to a reduction in such activities [34]. Other cognitive symptoms are tested by observing the presence of deficits in recognition memory using a Novel Object Recognition test [34], which could reflect the impairment due to the severity of the brain injury. Moreover, the aversive state of pain has been evaluated using the Conditioned Place Preference model and the Condition Place Aversion model. The Conditioned Place Preference model evaluates whether mCHI rodents, compared to sham controls, prefer spending more time in a chamber where a specific treatment is administered [34]. The Condition Place Aversion model assesses if drug-treated rodents no longer avoid chambers where a trigger was previously administered [34]. These behavioral models may not be sensitive enough to distinguish migraine from PTH from a phenotypical standpoint, even though they provide fundamental insight in the evaluation of the efficacy of specific treatments to alleviate headache-like symptoms in PTH. In this context, response to certain treatments could allow the elaboration of hypotheses on specific pathophysiological mechanisms that can be activated in PTH. Moreover, since certain PTH features such as cognitive impairment may also result from the brain injury itself, positive response to migraine treatments with the consequent application of *ex iuvantibus* criterion may further support their association with migraine rather than the exclusively underlying mTBI sequelae.

Concerning pathophysiological mechanisms, it is worth noting that the current models of PTH have disclosed specific pathways that can be present in primary headaches, such as migraine. *Bree* et al. showed persistent hypersensitivity to headache triggers in concussed animals [34], a finding that may explain persistent PTH and which is present as well in chronic migraine*.* In their study, 14 days after mTBI, when the mCHI-evoked cephalic hypersensitivity had disappeared, a systemic administration of low doses of GTN resulted in renewed cephalic tactile hypersensitivity, which could be attenuated by administration of sumatriptan or prevented by chronic treatment with murine anti-calcitonin gene-related peptide (CGRP) antibodies, started immediately after mTBI. Moreover, they showed a sumatriptan-induced conditioned place preference in mCHI animals, but not in sham controls. Considering also that triptans, migraine-specific acute medications, have presynaptic receptors that inhibit CGRP release, overall, these findings suggest that pain-related behaviors in the current mTBI model depend on peripheral CGRP.

In another study [41], cutaneous allodynia (both at periorbital and hind paw level) after mTBI was also attenuated by the administration of murine anti-CGRP antibodies, a fact that once again underlines a CGRP-dependent mechanism in acute PTH. Furthermore, they observed that early treatment after mTBI with anti-CGRP antibodies prevented the re-establishment of cutaneous allodynia after provocation with BLS, but a single administration right before BLS was not able to avoid its onset. These findings support the hypothesis that once central sensitization is established, this headache-related feature becomes independent from CGRP and may involve other mechanisms.

It seems that all the studies mentioned above strongly corroborate the hypothesis that common pathways, especially those involving CGRP, are present in both migraine and PTH, raising the question whether the two conditions could represent a continuum within a spectrum of headache disorders, especially considering that animal models of PTH have achieved reproducing migraine-like features, as seen in humans. On the one side, although being careful as data come from not specific PTH models, the existence of a link between PTH and migraine may be hypothesized; on the other, the same lack of specificity makes it difficult to identify the pathophysiological differences between the two disorders.

However, it is more likely that from distinct initial pathophysiology, some shared mechanisms are activated and converge in a similar subset of characteristics while other features may require the involvement of completely different pathways. For example, Navratilova et al. [41] showed that CGRP plays a major role in acute PTH and in promoting the transition to a persistent form of PTH, while it is probably less determining the symptoms once central sensitization is established, therefore once PTH has already become persistent. This fact seems to be partially different from chronic migraine patients, in whom, first, central sensitization is also present but together with elevated CGRP levels [43], and second, treatment with anti-CGRP antibodies is effective in chronic migraine [42, 44, 45]. Although chronic migraine animal models evaluating specific response to murine anti-CGRP drugs are lacking and human, and animal models are not directly comparable, the coexistence of other different mechanisms in chronic migraine and persistent PTH could still be supposed and should be further investigated.

Considering that no well-established animal models of PTH exist at present, the animal models mentioned above of mTBI displaying headache behaviors have helped the description of some of the pathophysiological pathways considered to be involved in PTH. Further studies need to be conducted to better investigate in PTH the role of certain mechanisms elicited in the study of mTBI and to assess their differential expression in migraine. For example, neurometabolic changes, where neuronal damage may be produced by glutamate release and an increase of extracellular potassium, have been shown in models of LFP concussive brain injury [46]. Other relevant mechanisms include neuroinflammation, as a consequence of glial cell activation after mTBI, as demonstrated in mCHI models that especially highlighted the role of mast cells [47]. At the same time, neuroinflammation seems to promote central nervous system excitability and therefore facilitates cortical spreading depression [48, 49], another pathway that has been observed in animal models of mTBI [39, 50] and implicated in the activation of the trigeminal sensory system.

Hereupon, it is fundamental to dispose of both PTH and migraine models and, although intrinsically different, applying similar measures and comparing them may represent a good strategy to detect similarities and differences in underlying mechanisms, therefore enabling further understanding of these disorders. For example, mechanisms related to cortical spreading depression could be studied, or features such as photophobia and its mechanisms can be investigated. Besides, considering as well that one of the most critical risk factors for developing PTH after mTBI is migraine [19], the study of both PTH and migraine, for example by reproducing mTBI in genetic migraine models, may provide further information, allowing comparison to exclusively migraine or PTH models.

At present, the preclinical study of PTH has to face other significant limitations. For example, current PTH animal models have shown impaired cognitive activities as well as altered responses to BLS, suggestive of migraine-like features as observed in human PTH studies [51]. PTH without migraine features is therefore not well studied, probably due to the lack of representative animal models that reproduce conditions similar to TTH, reflecting the fact that the underlying mechanisms of this disorder are still poorly understood. However, a recent study [51] on human PTH has shown different testing profiles in migraine-like PTH compared to TTH-like PTH, observing more cephalic and extracephalic thermal hypoalgesia that was accompanied by cephalic mechanical hyperalgesia in TTH-like patients. These findings should be further investigated in animal models of PTH with the objective of better characterizing these two subgroups and defining whether certain features, commonly tested, such as mechanical cephalic hyperalgesia are specific of one type or not.

Other questions, such as the multiple concussions and sub-concussive hits and their relation to PTH and its chronification have not been sufficiently addressed. Another issue is that the predisposition towards PTH in humans is not only associated with previous migraines but also with a history of psychiatric illnesses and comorbid psychiatric disorders [52], making it difficult to reproduce these aspects in animal models. All limitations considered, in the absence of better and well-established PTH models, at present mTBI animal models should still be considered useful in studying PTH when pain-related behavioral phenotypes indicative of headache are present.

### Pathophysiology

#### Do we need other models of head injury to study PTH?

Up to date, research in the field of PTH has not yet led to the full understanding of the disease and its successful treatment; it may be useful to understand whether changes in preclinical models help to improve their reliability and to promote the transfer from bench to bedside. The general pros and cons of TBI animal models are summarized in Table 2. As a premise, it is essential to consider that, although models of concussion, or mTBI as well as non-penetrating models are more relevant to PTH, some of the findings discussed below come from experimental models of more severe TBI and penetrating models. The inclusion of these latter models has been considered useful to give a comprehensive overview of all possible mechanisms that, in some way, may candidate to drive traumatic injuries to PTH.

**Table 2:** Pros and cons of animal models

Advantages	Disadvantages
- Precise control of physical parameters during trauma	- Differences in gross anatomy as compared to humans (e.g. lack of gyri/sulci)
- Trauma can be ‘dissected’ to focus on particular physical mechanisms, for example, rotational acceleration	- Differences in the physiology and timewise progression of pathology as compared to humans
- Possibility to control age and genetics (including sex)	- Few models for concussion available
- Possibility to monitor post-traumatically development of pathologies with exact timetables for evaluation and possibility to include baseline data	- Difficult to translate outcome parameters for concussion between rodents and humans

Over the last years, animal models have substantially improved and can now reproduce the different types of TBI, especially due to the more precise mechanical control on blast force and direction. Each model reproduces only one or two types of TBI similarly to human ones [53]. Given the heterogeneity of brain injury pathogenesis in humans, the characterization of biochemical and structural changes within each model and their comparative analysis may help to identify leading mechanisms of TBI and PTH better. Moye et al., in their review [54], summarized the most investigated mechanisms of PTH reproduced by different model systems of mTBI. The authors divided existing studies into two groups according to the types of observed molecules. The first group includes alterations in protein expression, specifically: i) 1. increased levels of CGRP in the trigeminal nucleus caudalis [55] and brainstem [39] in CCI model; ii) Toll-like receptor 4 (TLR4) impact on neuroinflammation pathways in weight-drop model with craniotomy and pituitary adenylate cyclase-activating polypeptide neuroprotective effect [56]; iii) brain-derived neurotrophic factor upregulation in spinal cord in LFP model [50]. Inflammatory mediators mostly represent the second group: i) increased level of TNF-α mRNA expression in injured cortex in CCI model [57]; ii) different activity of matrix metalloproteinases (MMP-2 and MMP-9) in close and open head injury models [58]; iii) potential role of dural mast cells degranulation in neuroinflammation and PTH in closed-head weight drop model and blast injury model [47].

Various observations showed relevant biological differences between animal models and humans. For example, it has been shown that between human and experimental animal astrocytes [59], there are morphological and functional differences, which in turn may lead to differences in secondary and tertiary changes after injury in humans and animals. Therefore, provisional modifications of the models should be aimed at reproducing the entire cascade of biochemical reactions associated with TBI in humans. It is safe to assume that early-stage in-vitro models may help to limit errors resulted from differences between humans and animals. For example, a model based on the blast impact (the compressed air-driven shock tube) on either the rodent neuroblastoma/glioblastoma hybrid cells or human neuroblastoma cells in tissue culture plates shows good prerequisites for studying primary, secondary and tertiary neurobiological changes TBI [60].

In order to perform appropriate modeling, it is necessary to underline the difference between PTH and TBI. Unlike TBI, which is defined by biochemical and biophysical tissue responses to trauma, PTH is a sensation, a subjective experience per definition. It follows that PTH modeling is impossible without assessing TBI, but it is also aimed at linking biochemical processes with behavioral responses. Several tests used for assessing pain perception, cognitive impairment, and depression in animals have been described [61]. However, as far as the clinical effects of TBI in humans are concerned, all these conditions may be superimposed in one person, whereas it is difficult to differentiate them by the behavioral tests in animal models. This complexity could be resolved, at least partly, via the collection of more data within the frameworks of the different models.

#### Sex differences in pain hypersensitivity

Migraine is a gendered disease, but gender/sex influence on PTH remains a grey area even if initial suggestions come from epidemiology [19–23]. Experimental studies suggest that increased estrogen levels may enlarge the receptive field of peripheral nociceptors in the trigeminal system [62]. Estrogens also affect the activity of the bradykinin B2 receptor and decrease the concentration of various neurotransmitters involved in its nociceptive pathways, such as substance P, glutamate, gamma-aminobutyric acid, dopamine, serotonin, and adrenaline [63, 64]. Estrogens and progesterone levels also affect the endogenous opioid system, mostly through μ-type of opioid receptors [65]. An experimental study in a rat model of PTH shows that females have an extended state of cephalic hyperalgesia, increased responsiveness to a headache trigger, and have a poorer response to anti-CGRP-therapy than males [66]. Notwithstanding these initial experimental premises and epidemiological findings that in humans suggest gender-driven differences, up to date, there are not enough data to draw a precise scenario about sex-related differences in PTH, in order to parallel it to migraine.

#### Is TBI necessary and sufficient or not?

Pre-existing migraine or tension-type headache (TTH) has been claimed as a predictor for the development of PTH [24], although no evidence exists [26]. A hypothesis to understanding the pathophysiology of persistent PTH is that TBI could ‘trigger’ or accentuate a TTH or a migraine pre-existing to the trauma [67]. Since in humans the most frequent traumatic injury associated with PTH is a mild closed head trauma, a concussion model evoked by a mild closed head injury has been developed [1]. The weight-drop concussion was associated with acutely enhanced processing of nociceptive signaling originating from trigeminal-innervated deep cranial tissues, due to meningeal mast cell degranulation [40]. These injuries/models did not affect central nociceptive processing that originates in extracranial tissues, indeed. Thus, in PTH it is plausible to hypothesize that direct trauma to the head may be enough to initiate acute periosteal and persistent dural mast cell degranulation for at least 30 days following a trauma, resulting in the development of headache [40, 47]. On the other side, Bree and Levy found that persistent activation of meningeal mast cells after mCHI is not required for the development of cephalic hypersensitivity, so that authors hypothesized that CGRP mediates the PTH-related pain behavior through a mechanism independent of ongoing meningeal mast cells pro-inflammatory response [68].

As mentioned above, direct involvement of CGRP in the pathophysiology of PTH has also been proposed. Multiple cortical spreading depression events, as can occur in the case of a TBI, upregulate in vivo CGRP mRNA levels at 24 h in the cerebral cortex of concussed rats, so that also CGRP levels can increase in discrete regions of the ipsilateral cortex when compared with contralateral cortex and the sham group (both ipsilateral and contralateral cortices) [69]. This study provides evidence for cortical spreading depression as a mechanism to initiate and maintain elevated CGRP levels in post-traumatic headache for a prolonged period [69]. Furthermore, other evidence comes from another study testing the administration of a novel anti-CGRP monoclonal antibody in a rat model of mCHI. Concussed rats developed cephalic tactile pain hypersensitivity that was ameliorated by sumatriptan or chronic blockade of CGRP using anti-CGRP monoclonal antibody starting immediately after mCHI and every six days subsequently. By two weeks, after the resolution of concussion-evoked cephalic hypersensitivity, the administration of glyceryl trinitrate produced a renewed and pronounced cephalic pain hypersensitivity that was again inhibited by sumatriptan or anti-CGRP antibody treatment [34].

An enhanced persistent susceptibility to migraine triggers could represent another mechanism for PTH. Still in the same rat model of mild concussive head injury, after the resolution of cephalic hypersensitivity, the administration of a low dose of GTN, acting as a migraine trigger, resulted in the re-exacerbation of cephalic tactile hypersensitivity up to 30 days post-injury as well as in a significant conditioned place aversion, having in reverse no effect in sham controls [34].

It is worth saying that current evidence, based on available models, does not exclude the involvement of peripheral damage to neck muscles, meninges, or other deep cranial tissues. The involvement of these tissues should be adequately investigated to define their differential role in PTH mechanisms, also to further understand if and how they overlap those of migraine [70–72].

## Conclusions

PTH is a common and disabling condition, for which we still need to clarify general pathogenesis and crucial mechanisms. Animal models have provided relevant information on the pathophysiology of PTH, but detailed underlying mechanisms are not fully understood. Importantly, recent data show a certain overlap with migraine, probably reflecting the involvement of some shared pathways. However, the evidence is currently extremely scarce. In order to better define the relation between migraine and PTH, and to improve specific knowledge that could lead to targeted treatments, animal models should be tailored to accurately resemble human features and be systematically used to seek similarities and differences between these two bothering conditions.

## Data Availability

Not applicable.
